# Identifying robust neural signatures of acupuncture modulation in healthy brains: a multimodal meta-analysis mapping core network

**DOI:** 10.3389/fnhum.2025.1494267

**Published:** 2025-09-02

**Authors:** Changhong Li, Yamin Liu, Baile Ning, Lihua Wu, Yuan Zhang, Junhe Zhou, Delong Zhang, Peng Zhou, Wenbin Fu

**Affiliations:** ^1^College of Teacher Education, Guangdong University of Education, Guangzhou, China; ^2^Shenzhen Bao’an Traditional Chinese Medicine Hospital, Guangzhou University of Chinese Medicine, Shenzhen, China; ^3^The Second Clinical College, Guangzhou University of Chinese Medicine, Guangzhou, Guangdong, China; ^4^Key Laboratory of Brain, Cognition and Education Sciences (South China Normal University), Ministry of Education, Guangzhou, Guangdong, China; ^5^School of Psychology, Center for Studies of Psychological Application, and Guangdong Key Laboratory of Mental Health and Cognitive Science, South China Normal University, Guangzhou, China

**Keywords:** multimodal neuroimaging, acupuncture, ALE meta-analysis, functional connectivity, pain processing

## Abstract

Acupuncture, a cornerstone of Chinese integrative medicine, demonstrates clinical efficacy for pain- and inflammation-related conditions through extensive clinical practices. Using multimodal neuroimaging techniques (fMRI and PET), numerous studies have investigated acupuncture’s impact on brain structure and function. However, a systematic integration of these findings is still lacking. To address this issue, we conducted a meta-analysis using the activation likelihood estimation (ALE) approach to identify brain regions that consistently modulated by verum acupuncture in both healthy populations and patients. Critically, establishing neural signatures in healthy populations provides a foundational baseline for normative brain responses, which is essential to detect pathological deviations in patient cohorts. Subsequent behavioral domain analyses elucidated the functional profiles of these significant clusters. To characterize whole brain functional connectivity architecture that associated with identified regions, we employed meta-analytic connectivity modeling (MACM) and seed-based resting-state functional connectivity (rsFC) analyses. Our analyses consistently implicated the bilateral inferior parietal lobule (extending into postcentral gyrus) and right thalamus, key regions within the somatosensory pain processing network, as core neural substrates that modulated by acupuncture in healthy populations. Functional connectivity analyses further demonstrated that these brain clusters engaged a distributed pain-processing network, including bilateral supplementary motor area (SMA), superior temporal gyrus (STG), and insular cortex. Taken together, these findings provide a mechanistic neurobiological basis for acupuncture-induced pain processing and identify potential therapeutic targets within the pain-associated neural circuitry.

**Systematic review registration:**

https://www.crd.york.ac.uk/PROSPERO/view/CRD42024562566, identifier CRD42024562566.

## 1 Introduction

Acupuncture, a complementary therapy that rooted in traditional Chinese medicine, demonstrates significant clinical potentials in modulating physiological functions and alleviating symptoms across diverse disorders (Kaptchuk, 2002). This core procedure involves inserting needles into specific anatomical points (acupoints), producing measurable therapeutic effects, particularly analgesia (Lin and Chen, 2008). The therapeutic foundation of acupuncture rests on stimulating specific acupoints along meridian pathways to regulate corresponding organ functions and systemic homeostasis. Clinically, the sensory sensation known as “*Deqi*,” that usually characterized by descriptors such as soreness (suan), numbness (ma), distension (zhang), and pain (tong), is often considered indicative of effective needle manipulation and physiological engagement (Yang et al., 2013). To enhance therapeutic efficacy, traditional manual needle manipulation frequently incorporate some adjunctive techniques like electroacupuncture or laser stimulation (Jun et al., 2015). Despite its established clinical utility, the precise neural mechanisms underpinning acupuncture’s modulation of sensory processing and broader therapeutic effects remain incompletely understood, particularly in pain management.

Given acupuncture’s capacity to modulate brain activity, it serves as a valuable tool for probing neural mechanisms using non-invasive functional imaging techniques (Cao et al., 2020; Dhond et al., 2007). Notably, functional magnetic resonance imaging (fMRI), particularly resting-state (rs-fMRI) which captures intrinsic brain dynamics without task demands, is capable to characterize acupuncture-induced neural responses in both healthy populations and patients (Cai et al., 2018; Feng et al., 2011; Hui et al., 2009). Compared to sham acupuncture, verum acupuncture in healthy populations typically elicited activation in several pain-processing regions (e.g., insular, thalamus, anterior cingulate cortex, postcentral gyrus, and supplementary motor cortex) and hypoactivation in limbic and default mode network (Chae et al., 2013). In clinical practice, patients with comorbid psychiatric and neurological disorders frequently suffer from refractory pain syndromes, including depression-associated pain, migraines and post-stroke pain. Previous neuroimaging studies (Lin et al., 2025; Qian et al., 2025; Wu et al., 2017) found that verum acupuncture-induced neural modulations were primarily centered in sensory regions and insular, while heterogeneous activity in other brain regions might reflect pathological signatures with specific diseases. Consistent with fMRI findings, positron emission tomography (PET) demonstrated congruent regional perfusion and metabolic changes within these brain areas (Huang et al., 2007; Lai et al., 2009; Yang et al., 2014). Therefore, these findings indicate a core set of brain regions, which is critical for processing acupuncture’s somatic effects and mediating its broader physiological regulation. For instance, the insular cortex acts as an integrative hub linking sensory inputs with autonomic networks for homeostatic control. The thalamus and postcentral gyrus are fundamental for transforming peripheral inputs into consciously perceived perception (Rao et al., 2024). However, bridging acupuncture’s clinical efficacy with the inconsistently neural substrates remains a critical barrier to its mechanistic understanding. Substantial heterogeneity across studies in populations (healthy vs. patient), experimental designs (task vs. rest, verum vs. sham), as well as methodological approaches, has obstructed the identification of reproducible neural signatures supporting acupuncture’s core mechanisms.

To establish robust neural signatures of acupuncture, we conducted the activation likelihood estimation (ALE) meta-analysis approach for quantifying spatial convergence across neuroimaging studies (Eickhoff et al., 2009, 2012). ALE statistically models reported peak coordinates (foci) from individual neuroimaging studies as spatial probability distributions. Significant spatial convergence of foci across studies is identified by comparing the modeled results against an empirically derived null distribution of random spatial association. This method has demonstrated robust reliability in synthesizing spatially heterogeneous neuroimaging findings across cognitive, affective, and clinical domains. Although prior meta-analyses have examined acupuncture’s neural correlates of acupuncture (Huang et al., 2012; Park et al., 2017), the evolving literature necessitates an updated synthesis by incorporating contemporary findings. In this current study, we hypothesize that specific brain circuits, particularly within the central pain-processing network, exhibit sufficient reproducibility across independent datasets and may serve as candidate biomarkers underlying acupuncture’s therapeutic mechanism. To address this gap and test our hypothesis, we employ ALE meta-analysis to systematically identify consistent brain regions modulated by verum acupuncture across published fMRI and PET studies in both healthy individuals and patient populations.

## 2 Materials and methods

### 2.1 Literature search and study criteria

Neuroimaging studies utilizing acupuncture interventions were identified through systematic literature searches in PubMed, Web of Science, and Scopus. The search strategy employed combinations of the following key terms: “functional magnetic resonance imaging,” “acupuncture,” and “positron emission tomography.” After removing duplicates, 170 neuroimaging studies were retained for further analysis. Inclusion criteria were defined as follows:

(1)    The paper was written in English and strictly reviewed before publication.
(2)    Multimodal acupuncture approaches were included, such as traditional needle acupuncture, electroacupuncture, and laser acupuncture.
(3)    Acupoints must be conducted in human subjects aged at least 18 years instead of animal models or computational stimulations.
(4)    During statistical analysis, whole brain voxel-wise comparison is necessary to extract the specific coordinates for each experiment. Thus, studies using region-of-interest (ROI)-based analysis or functional connectivity were not taken into consideration.
(5)    Study contrasts contained two types: pretreatment vs. posttreatment and sham control vs. real acupuncture.
(6)    Significant findings of neuroimaging research were reported as 3D coordinates in Talairach or Montreal Neurological Institute (MNI) space.

**FIGURE 1:**
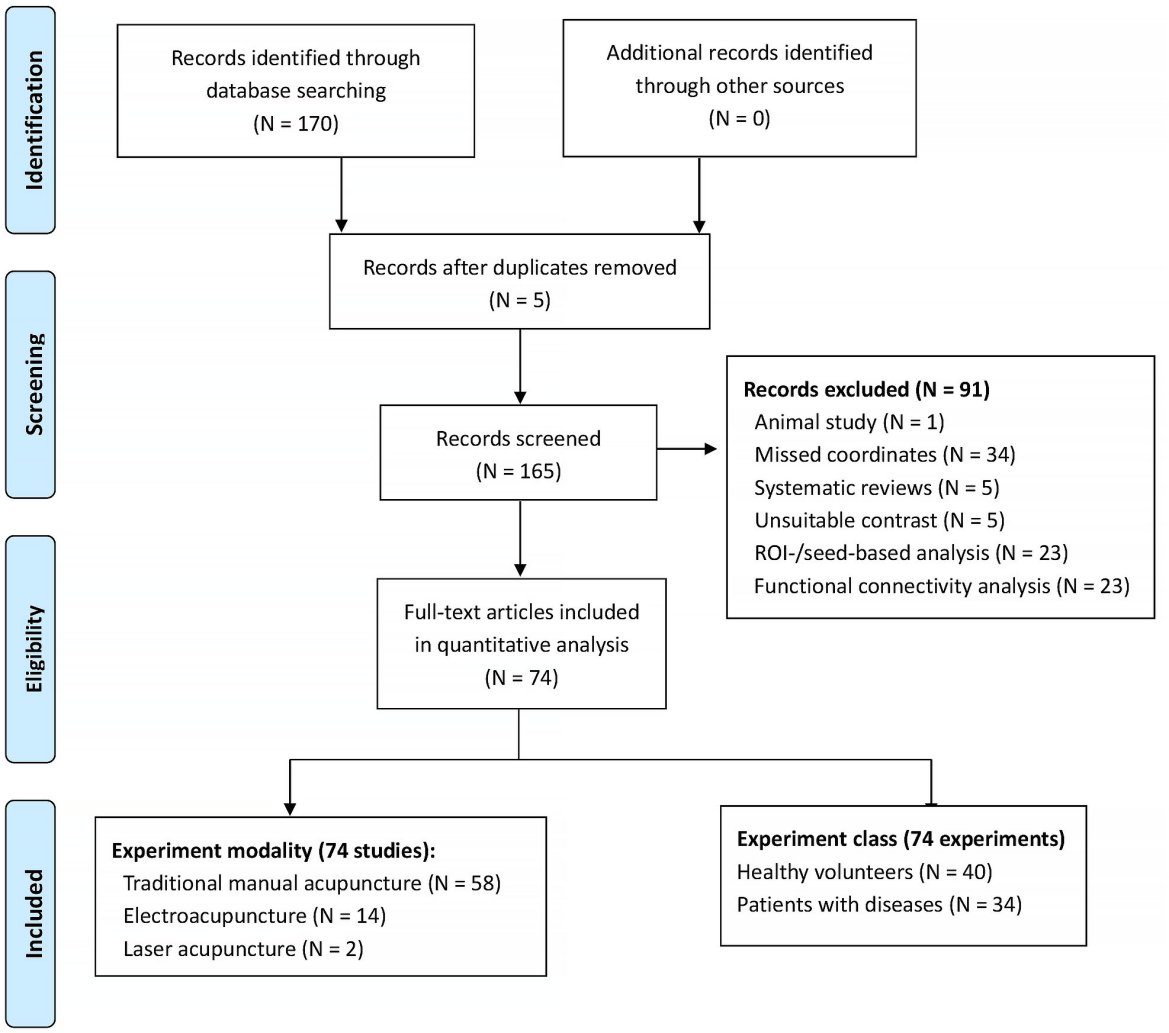
Flowchart of meta-analytic neuroimaging literatures review. ROI, region-of-interest.

Given potential divergences in acupuncture’s neuroimaging effects between healthy populations and clinical cohorts, we conducted separate literature searches for each group. The study encompassed diverse clinical conditions, such as ischemic stroke, migraine, depression, and chronic pain syndromes. For each experiment, the statistical threshold was set at *p* < 0.05 level. Multiple comparisons correction was not mandatory and depended on the study. After excluding those studies without any coordination information, we extracted the coordinates for each experiment for further analysis. For the same neuroimaging datasets with different statistical approaches, we combined their coordinates to avoid potential statistical bias. The literature review was finalized by the end of June 2024. This review study has been registered at PROSPERO (CRD42024562566). Figure 1 shows the basic flowcharts of our literature research. For the detailed characteristics of the included literatures, please refer to Supplementary Table 1 in the supplemental materials.

### 2.2 ALE analysis

In this study, we applied ALE meta-analysis approach to examine spatial convergences over all of the foci (that is, the coordinates) across neuroimaging studies, which was computed using GingerALE3.0.2. This approach calculates spatial convergence of activation foci against a null hypothesis of random spatial distribution, thereby identifying statistically significant clusters within distinct brain areas. Firstly, the ALE model estimation generated a modeled activation (MA) map that was built by the Gaussian distributions. The full-width half-maximum (FWHM) was determined by the sample size of corresponding experiment. Subsequently, we calculated the union of the MA maps across all inclusion experiments for each voxel and analyzed the significances using a cluster-thresholded approach. Consistent with *a priori* ALE study (Muller et al., 2018), we determined a significance threshold was set at a cluster-level inference of *p* < 0.05 and a cluster-forming threshold of *p* < 0.001. To address potential confounds from experimental paradigms, we conducted independent ALE meta-analyses for two subgroups: sham acupuncture vs. real acupuncture, and pre-acupuncture vs. post-acupuncture conditions. In addition, we methodically excluded 4 non-fMRI studies in healthy populations and 3 non-fMRI studies in patient cohorts for resolving the modality-specific differences between PET and fMRI findings.

### 2.3 Behavioral domain (BD) analysis

Following ALE analysis, the operation functions of each significant brain cluster was identified using behavioral domain analysis. This behavioral system is classified into six primary domains: cognition, action, perception, emotion, interoception, or pharmacology. We utilized the Brain Annotation Toolbox (Liu et al., 2019) to identify those functional profiles of significant clusters based on Neurosynth activation maps (Yarkoni et al., 2011). The mode of permutation analysis was settled as “clusters” and number of permutation times was 1000. The brain functions significantly associated with the given ROIs at *p* < 0.05 level.

### 2.4 Meta-analytic connectivity modeling (MACM) approach

We performed meta-analytic connectivity modeling (MACM) approach to characterize whole-brain co-activation profiles for each significant cluster derived from ALE analysis (Laird et al., 2009). Using this technique, a well-known meta-analysis of whole brain functional connectivity patterns with the amygdala was conducted (Robinson et al., 2010). Using the peak coordinates of each significant brain cluster, we created spherical regions of interest (ROIs) with an 8-mm diameter. Then, we searched all experiments that discovered increased activation (that is, Context-Normal Mapping, Activation-Activations only, and Diagnosis-Normals) for each ROI. Given the outcomes of our behavioral domain analysis, we restricted the task-related condition to Somesthesis-Pain in Perception. The whole brain coordinates in matching-search criteria literature were downloaded from the BrainMap database (Laird et al., 2005). By using GingerALE3.0.2, significant convergences of functionally connected regions were identified at a cluster-level inference of *p* < 0.05 and cluster-forming threshold of *p* < 0.001 (Eickhoff et al., 2017).

### 2.5 Seed-based functional connectivity (FC) analysis in healthy populations

To reproduce the whole-brain FC patterns in real resting-state fMRI datasets, we used Zang’s public datasets from the 1000 Functional Connectomes Project^1^ to perform the seed-based FC analysis. We totally recruited 185 young volunteers (70 males, age: 21.19 ± 1.84 years old). Using a 3T MRI scanner, both a resting-state fMRI dataset (matrix size = 64 × 64 mm^2^, voxel size = 3.125 × 3.125 × 3.6 mm^3^, TR = 2 s, number of slices = 33, 225 volumes) and 3D T1-weighted structural images were archived. We preprocessed the rs-fMRI images by using DPABI_v6.0 (Yan et al., 2016) and SPM12. The main procedures included: first 10 volumes were discarded, Slice timing, Realignment and motor correction (exclusion criteria: head transitions < 1.5 mm, rotations < 1.5°, or mean Framewise Displacement < 0.27 mm), Covariances regression (Friston 24-motion parameters, signals from cerebrospinal fluid, and signal from white matter), Spatial normalization in MNI standard space by DARTEL method, Smooth with 8 mm^3^ FWHM, Linear detrend, and Bandpass filtering (0.01∼0.08 Hz). Then, we selected the ALE-derived cluster as the seed and calculated functional connectivity with other voxels over the whole brain. After normalizing all the FC maps, one-sample *t*-tests were applied to examine significant brain clusters (significant level at *p* < 0.01 with Bonferroni correction).

## 3 Results

### 3.1 ALE analysis

**FIGURE 2:**
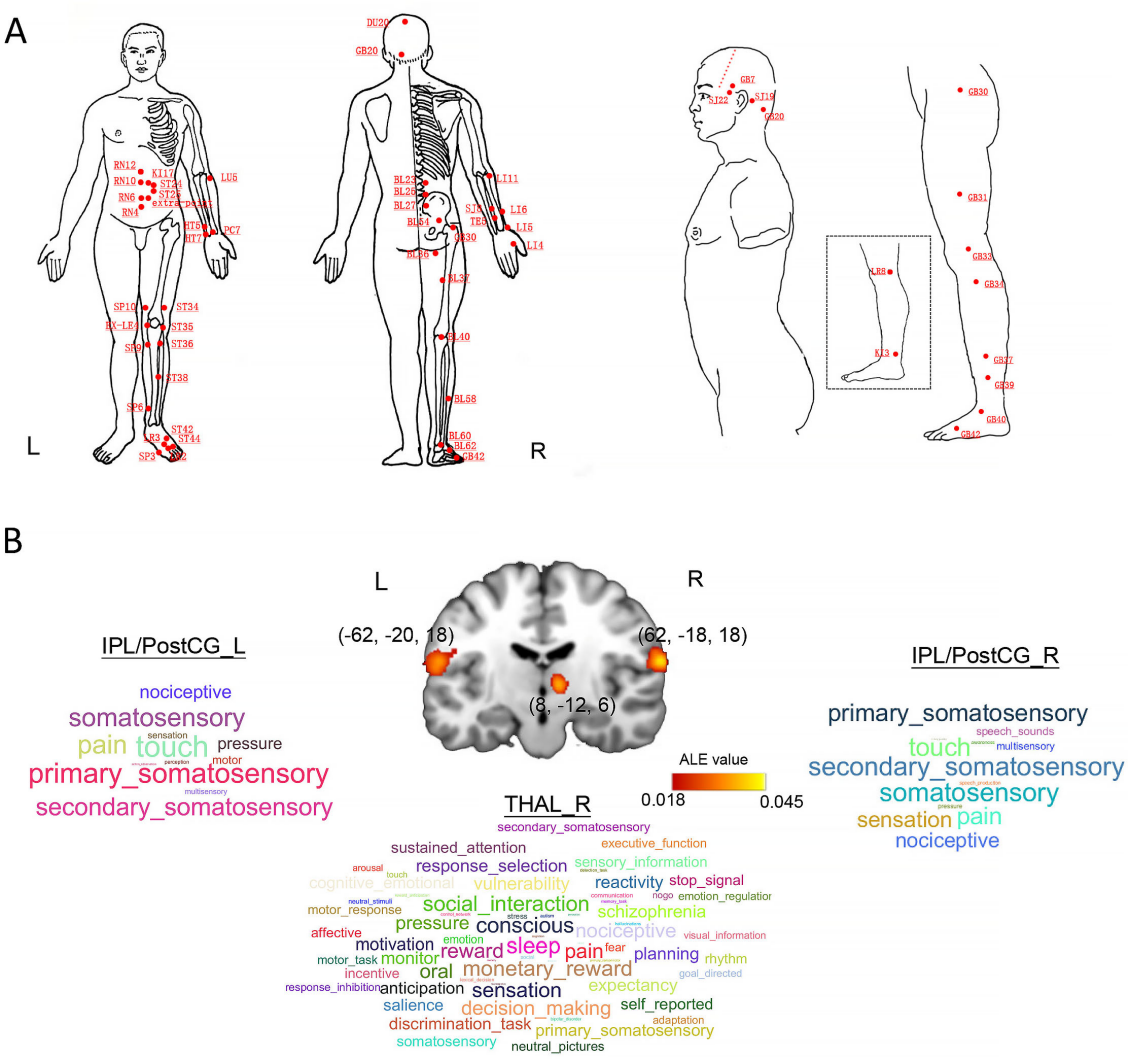
Locations of acupoints, statistical results for the ALE meta-analysis study, and behavioral domain (BD) analysis. **(A)** All of the relevant acupoints in those selected literatures. **(B)** Visualized demonstration of our ALE meta-analysis and corresponding behavioral domains for each brain cluster. CV4, Guanyuan; CV6, Qihai; CV10, Xiawan; CV12, Zhongwan; KI17, Shangqu; ST24, Huaroumen; ST25, Tianshu; HT5, Tongli; HT7, Shenmen; LU5, Qi-ze; PC7, Daling; SP10, Xuehai; EX-LE4, Neixiyan; SP3, Taibai; SP6, Sanyinjiao; SP9, Yinlingquan; ST34, Liangqiu; ST35, Dubi; ST36, Zusanli; ST38, Tiaokou; ST42, Chongyang; ST44, Neiting; LR2, Xingjian; SP3, Taibai; BL23, Shenshu; BL25, Dachangshu; BL27, Xiaochangshu; BL36, Chengfu; BL54, Zhibian; BL37, Yinmen; BL40, Weizhong; BL58, Feiyang; BL60, Kunlun; BL62, Shenmai; GB42, Diwuhui; LR8, Ququan; KI3, Taixi; GB31, Fengshi; GB33, Xiyangguan; GB34, Yanglingquan; GB37, Guangming; GB39, Xuanzhong; GB40, Qiuxu; GB42, Diwuhui; TE22, Erheliao; GB7, Qubin; TB19, Sanyangluo; GB20, Fengchi. IPL/PostCG, inferior parietal lobule/postcentral gyrus; THAL, thalamus.

Finally, we separately identified 39 studies (38 experiments, 723 subjects, and 1032 foci) in the healthy populations and 28 studies (32 experiments, 918 subjects, and 434 foci) in the patients. We only found that the bilateral inferior parietal lobule/postcentral gyrus (IPL/PostCG, BA40, peak: 62, −18, 18 and −62, −20, 18) and right thalamus (THAL, peak: 8, −12, 6) were significantly convergent in the healthy participants (Figures 2A, B). For the healthy populations, there were 16 experiments comparing sham and real acupuncture conditions and 22 experiments utilizing pre-post or block design methodologies. However, we did not observe any significances in the patients with a variety of diseases. For the sham and real acupuncture comparison, our analysis revealed significantly convergent activity in the bilateral IPL/PostCG (peak: 64, −16, 16 and −60, −18, 18) and right thalamus (peak: 8, −12, 6). In the pre-post subgroup, significantly convergent activities were localized to the right IPL/PostCG (peak: 64, −18, 18). These finding robustly align with our core findings (IPL/PostCG and thalamus) that derived from sham-controlled experimental design in the healthy populations. Moreover, our results only revealed significant convergences of neural activity in the bilateral IPL/PostCG (peak: 62, −22, 22 and −62, −20, 22) and right thalamus (peak: 8, −14, 4) for the fMRI datasets of healthy subjects.

### 3.2 Behavioral domain results

We identified that the right IPL/PostCG and left IPL/PostCG were significantly associated with 12 and 13 functions with a significant level at *p* < 0.05, such as pain, sensation, perception, and primary somatosensory (Figure 2B). Similarly, Figure 2B also presents that the right thalamus is significantly correlated with 66 functions, including pain, consciousness, nociceptive, and sensation.

### 3.3 MACM results

**FIGURE 3:**
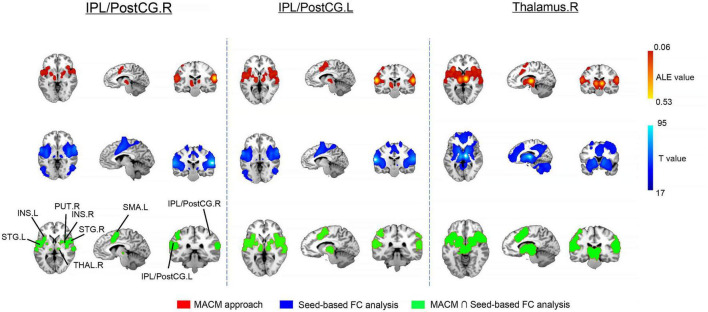
Results of meta-analytic connectivity modeling (MACM) and whole brain seed-based functional connectivity (FC) analysis in healthy participants. For the latter analysis, we only retained 80% top for the significant brain clusters. IPL/PostcG, inferior parietal lobule/postcentral gyrus; INS, insular; SMA, supplemental motor areas; THAL, thalamus; STG, superior temporal gyrus; PUT, putamen; R, right; L, left.

After searching the eligible neuroimaging literatures, our study acknowledged 108 experiments with 1590 participants for the right IPL/PostCG, 189 experiments with 2757 participants for the left IPL/PostCG, as well as 391 experiments with 6031 participants for the right thalamus. Using the MACM approach, we detected some common regions that were significantly coactivated, including the bilateral supplemental motor areas, bilateral superior temporal gyrus, bilateral putamen, and bilateral insular (Figure 3).

### 3.4 Seed-based whole brain FC analysis

For all ROIs, our statistical maps were thresholded at *p* < 0.01 with Bonferroni correction and further reported widely distributed brain clusters. Noticeably, the bilateral supplemental motor areas, bilateral putamen, and bilateral insular that functionally connected with the bilateral IPL/PostCG, and right thalamus in the healthy participants as well (Figure 3).

## 4 Discussion

Our ALE meta-analysis establishes robust neural substrates underlying verum acupuncture, revealing consistently convergent patterns across neuroimaging studies. Crucially, significant clusters within the bilateral IPL/PostCG and right thalamus emerged as core neural signatures modulated by acupuncture in healthy populations. Behavioral domain analysis revealed that these brain regions are functionally specialized for somatosensory processing with distinct roles in pain perception, nociception, and multisensory integration. Importantly, MACM revealed significant functional coupling between these key regions (the bilateral IPL/PostCG and right thalamus) and critical hubs of the central pain-processing network. This interconnected circuitry primarily encompassed the bilateral SMA, STG, insular, and putamen. Moreover, this functional network architecture was validated independently by whole brain seed-based FC analysis using rs-fMRI dataset from 185 healthy young adults. To sum up, these convergent multimodal findings highlight the bilateral IPL/PostCG and right thalamus as pivotal neural substrates. These regions orchestrate a distributed but functionally integrated network underlying acupuncture’s effects, providing a mechanistic framework for investigating acupuncture-induced pain processing and advancing its clinical translation. While informative for elucidating the neurophysiological foundations of acupuncture, these findings may not directly translate to clinical analgesic mechanisms due to the lack of significant convergence in patient-derived neuroimaging datasets. Future research contrasting neural responses between healthy controls and clinical cohorts will be essential to identify therapeutic-specific neural substrates.

Following verum acupuncture, our meta-analysis robustly identified the bilateral IPL/PostCG and right thalamus as core targets in healthy individuals. This consistent neural activity establishes their role in mediating acupuncture’s modulatory effects on somatic sensation, particularly pain perception. Of note, neuroimaging studies (Jin et al., 2018; Li L. L. et al., 2018; Nierhaus et al., 2015) targeting major limb acupoints (e.g., Sanzuli, Sanyinjiao, and Tongli) reliably engage these brain areas. Behavioral domain analysis further clarifies that the bilateral IPL/PostCG sustains somesthetic awareness and directs spatial attention to bodily stimuli, potentially induced by self-reported *deqi* sensation during needle manipulation. The IPL’s specialization in mediating spatially-directed bodily attention is well-established (Jung et al., 2015; Vandenberghe et al., 2001) and aligns with our findings. Crucially, this neural activity pattern was failed to reproduce in our patient populations, revealing disease-specific modulation of acupuncture’s neural targets. Although several fMRI studies have reported IPL/PostCG engagement in patients (Cho et al., 2013; Li Z. et al., 2018; Tan et al., 2017), the absence of significant spatial convergence in our meta-analysis suggests fundamentally distinct pain regulatory mechanisms in clinical populations. We hypothesize that this divergence may arises from dysfunctional attentional allocation toward somatic signals or reorganized functional integration within core pain-processing circuits in patients. As the key hub in sensory pathways, the thalamus gates conscious pain perception and dynamically facilitates sensory transmission to cortical regions (Chen et al., 2014). In patients, acupuncture modulated thalamocortical functional connectivity associated with motor-related brain areas (Li Z. et al., 2018; Xie et al., 2014), suggesting adaptation of neurocircuitry for therapeutic analgesia. In summary, our findings suggest that verum acupuncture in healthy individuals consistently modulates neural activity within the bilateral IPL/PostCG and right thalamus, which are core hubs for somatic sensory processing and allocations of bodily attention.

Our MACM and seed-based FC analyses converged to reveal a distributed brain network with key hubs in the bilateral IPL/PostCG and right thalamus, exhibiting extensive functional connectivity to the insular, SMA, and STG. This circuit subserves integrated sensory processing, pain modulation, as well as higher-order cognitive evaluation of bodily state awareness (Duerden and Albanese, 2013; Legrain et al., 2011; Price, 2000; Tian et al., 2025). In addition, this functional network architecture demonstrates significant reproducibility across neuroimaging studies employing diverse acupuncture protocols (Jung et al., 2015; Theysohn et al., 2014). Within this circuitry, the insular serves as a pivotal integration hub, synthesizing sensory inputs with cognitive and affective processes to form an interoceptive representation of bodily states (Kurth et al., 2010). As demonstrated in our study and existing literatures, the insula serves as a core node within the pain matrix in healthy individuals, which facilitates pain integration and interoceptive processing (Kong et al., 2006; Simone et al., 2025). In patients with chronic low back pain, investigator observed that low-frequency oscillations in the insula were associated with immediate pain alleviation following ankle acupuncture (Xiang et al., 2019). By synthesizing these findings with prior evidence (Lai et al., 2025; Wen et al., 2021; Zyloney et al., 2010), we propose that acupuncture stimulation may alter the brain responses to pain stimuli by interfering with the functional connectivity between insular and other brain region. Further research remains necessary to validate this hypothesis. Supplementary motor area, a key region for motor planning and pain modulation, was activated by stimulating specific acupuncture points (Han et al., 2019). Therefore, our findings delineate a core large-scale whole brain functional architecture that consistently modulated by verum acupuncture with diverse protocols in the healthy populations. This network functionally integrates sensory-discriminative components (IPL/PostCG and thalamus), affective-cognitive regions (insular and STG), and motor-sensory gating (SMA), providing a neurobiological framework for acupuncture’s simultaneous modulation of pain perception and somatic awareness.

Given the spatial convergence of brain regions, our findings further identify attentional modulation as a plausible central mechanism underlying the acupuncture’s therapeutic effects. This interpretation is grounded in the well-established Tripartite Attention Network Framework, encompassing alerting, orienting, and executive control systems (Posner and Dehaene, 1994). Specifically, the alerting network facilitates rapid detection of salient stimuli by transiently suppressing the executive control network, thereby prioritizing sensory and cognitive processing of relevant events (Callejas et al., 2004; Posner and Dehaene, 1994). That is, it monitors threats to bodily integrity and coordinates defensive responses to noxious stimuli (Apkarian et al., 2005; Reddan and Wager, 2018; Legrain et al., 2011). As core acupuncture targets, we propose that right IPL acts as a key neural substrate for sustaining attentional focus on salient somatic states. Therefore, we hypothesize that acupuncture-induced activity in this key region is to mediate the dynamic coordination between the alerting and executive control attentional systems. This mechanism of inter-network attentional recalibration provides a unified neurocognitive model for acupuncture’s clinical efficacy, particularly its capacity to normalize threat-response patterns to bodily signals through dynamic network reconfiguration (Corbetta and Shulman, 2002; Sarter et al., 2001). Nevertheless, further empirical investigation is required to characterize this mechanism across heterogeneous populations.

Though our findings suggest a consistent neural signature for acupuncture’s effect, several limitations merit rigorous consideration. First of all, substantial methodological variability across neuroimaging studies introduces inherent clinical and neurobiological heterogeneity, including diverse acupoint selections, treatment modalities, patient populations, and control conditions. These heterogeneities impede the clinical translatability of our results and underscore the need for mechanistic stratification in future neuroimaging protocols. Secondly, our meta-analysis synthesized evidences from 32 experiments reporting 434 coordinate foci in the patients. While the ALE methodology is robust to moderate sample sizes, the statistical power still remains constrained for detecting disease-specific effects. Larger-scale prospective neuroimaging studies employing standardized designs are essential to replicate findings and improve effect size estimation. Longitudinal neuroimaging protocols integrating chronic treatment schedules and precise temporal sampling are imperative to elucidate the neuroplastic mechanisms mediating acupuncture’s therapeutic effects. In the end, the inclusion of heterogeneous acupuncture modalities prevents definitive attribution of network modulations to core neurobiological mechanisms owing to the potential technique-specific artifacts. Consequently, we should systematically resolve these variabilities through strict coordinate inclusion criteria and comprehensive subgroup analyses. These limitations delineate essential directions for future research. Future studies integrating mechanism-driven experimental designs, larger samples, temporal profiling, and modality comparisons are needed to transform our neural signatures into clinical translation of acupuncture’s effects.

## 5 Conclusion

In summary, our findings map consistent neuroanatomical responses to verum acupuncture stimulation in healthy individuals, primarily involving the bilateral IPL/PostCG and right thalamus. These regions are implicated in somatosensory processing and may reflect neural encoding of needling perception rather than disease-modifying effects. Furthermore, we characterized a distributed network that underlies the pain modulation, including bodily signal processing, pain modulation, and allocation to bodily states. Critically, these patterns represent acute neurophysiological responses in pain-free subjects and should be further validated therapeutic efficacy for clinical pain. The absence of convergent fMRI findings in patient cohorts underscores that acupuncture’s putative analgesic mechanisms in pathological states remain unconfirmed by this study.

## Data Availability

The raw data supporting the conclusions of this article will be made available by the authors, without undue reservation.

## Footnotes

1
http://fcon_1000.projects.nitrc.org/fcpClassic/FcpTable.html
