# Supplementary material for: Identifying robust neural signatures of acupuncture modulation in healthy brains: a multimodal meta-analysis mapping core network
Source: Front Hum Neurosci. 2025 Sep 2;19:1494267. doi: 10.3389/fnhum.2025.1494267 (PMC12436331; doi:10.3389/fnhum.2025.1494267)
Supplement: Supplementary file 1 [file Table_1.docx]

Table S1 Experimental details of all 66 meta-analytic neuroimaging studies.

| Authors | Study design | Number of subjects and Age | Interventions and acupoints | Imaging modality | Normalizing Software | Reported standard space |
| --- | --- | --- | --- | --- | --- | --- |
| **Heathy subjects** | | | | | | |
| An et al. (2009) | Pre - post acupuncture | SPECT: 20 (17 men, ages 24–31 years);  ^18^F-FDG PET: 13 (12 men, ages 23–30 years). | Electroacupuncture at two Hegu and Quchi (LI 4 and LI 11 in the right upper) for15 mins | SPECT and  ^18^F-FDG PET/CT | SPM2 | MNI |
| Lai et al. (2009) | Sham - true acupuncture | 18 (9 men, ages 22-27 years) | Manual acupuncture at Waiguan (TE5) for 15 mins | ^18^F-FDG PET/CT | SPM2 | Talairach |
| Biella et al. (2001) | Sham - true acupuncture | 13 young men, ages 21–32 years | Manual acupuncture at Zusanli (ST 36) and Qi-ze (LU 5); Sham acupuncture at points 1 cm lateral to the two acupoints for 25 mins | H_2_^15^O bolus PET | SPM1996 | Talairach |
| Wang et al. (2007) | True - Non acupuncture | 14 (9 men, ages 22-27 years) | Electroacupuncture at right Hegu (LI 4, 9 subjects) and facial non-acupoint points (5 subjects) | BOLD fMRI | SPM2 | Talairach |
| Fang et al. (2009) | Sham - True acupuncture | 10 (5 men, ages 22-28 years) | Manual acupuncture at Taichong, Xingjian, and Neiting (LR3, LR2, and ST44) and a sham point for 6 mins | BOLD fMRI | SPM99 | MNI |
| Jiang et al. (2013) | Block-designed study | 18 (9 men, mean age 22 years) | Manual acupuncture, electroacupuncture and transcutaneous electrical acupoint stimulation and sham in Zusanli (ST 36) on the left leg | BOLD fMRI | SPM5 and GIFT | MNI |
| Long et al. (2016); Nierhaus et al. (2015) | Sham - True acupuncture | 22 (11 men, ages 21-32 years) | Manual acupuncture at Zusanli (ST 36) and two control acupoints for 6 mins | BOLD fMRI | SPM8 | Talairach |
| L. L. Li et al. (2018) | Sham - True acupuncture | 20 (10 men, ages 22-28 years) | Electroacupuncture at Tongli (HT 5) and a sham acupoint for 8 mins | BOLD fMRI | SPM8 | Talairach |
| Jin et al. (2018) | Block-designed study | 30 (ages 20-25 years) | Manual acupuncture at Sanyinjiao (SP 6) on the right side of the body | BOLD fMRI | SPM8 | Talairach |
| A. Li et al. (2018) | Pre - post acupuncture | 30 (15 men, mean age 24.3 years; 15 women, mean age 24.6 years ) | Manual acupuncture at Liangqiu (ST 34), Xuehai (SP 10), Neixiyan (EXLE4), and Dubi (ST 35) for 8 mins | BOLD fMRI | SPM12 | MNI |
| Liu et al. (2018); (Liu et al., 2016) | Pre - post acupuncture | 10 (4 men, ages 20-34 years) | Manual acupuncture at left Yanglingquan (GB 34) for 15 mins | BOLD fMRI | SPM8 and DPARSF | MNI |
| Wang et al. (2021) | Sham - True acupuncture | 15 (7 men, 28.0 ± 4.24; 8 women, 27.4 ± 3.65) | Manual acupuncture at Taibai (SP 3) for 15 mins | BOLD fMRI | DPARSF | MNI |
| Yang et al. (2017) | Block-designed study | 34 (21 men, 22.09 ± 1.26 years) | Manual acupuncture at Shenmen (HT 7; 9 males, 8 females) or Yangxi (LI 5; 12 males, 5 females) | BOLD fMRI | SPM8 | Talairach |
| He et al. (2020) | Pre - post acupuncture | 20 healthy young (9 men, ages 24.7 ± 2.9), 20 healthy elders (8 men, age 56.8 ± 7.2) | Manual acupuncture at Taixi (KI 3) for 3 mins | BOLD fMRI | DPARSF | MNI |
| Cao et al. (2020) | Sham - True acupuncture | 24 (8 men, ages 25.2 ± 0.8 years) | Manual acupuncture at right Sanyinjiao (SP 6) and Yinlingquan (SP 9) for 20 mins | BOLD fMRI | CONN and CAT12 | MNI |
| Quah-Smith et al. (2013) | Block-designed study | 16 (mean age 48.2 years ) | Laser acupuncture at Ququan (LR8) and needle acupuncture in Ququan (LR8) on the other leg | BOLD fMRI | SPM2 | MNI |
| Yoo et al. (2004) | Sham - True acupuncture | 12 (7 men, aged 27.2 F 6.3, all right-handed) | Manual Acupuncture at PC6 point of the right wrist | BOLD fMRI | SPM99 | Talairach |
| Yoo et al. (2007) | Sham - True acupuncture | 12 (three females, mean age = 25.2 ± 6.5) | Manual Acupuncture at LI4 | BOLD fMRI | SPM2 | Talairach |
| Hui et al. (2009) | Block-designed study | 48 (mean=28.6±7.56 SD, 19 M, 29F) | Manual Acupuncture at LI4, ST36 and LV3 | BOLD fMRI | AFNI | Talairach |
| Kong et al. (2007) | Block-designed study | 8 (4 males,  mean age 29±7 years) | Manual Acupuncture at three points (UB 60, GB 37, and a non-acupoint) on the right leg | BOLD fMRI | SPM2 | MNI |
| Chae, Lee, Kim, Sohn, et al. (2009) | Block-designed study | 10 (6 men, 4 women; age range 20–34 years). | Manual acupuncture at acupoint LR2 | BOLD fMRI | SPM5 | Talairach |
| Li et al. (2008) | Single block design | 53 (31 men), aged 23.7 3.6 years | Manual acupuncture at LR3, Taichong; LR6, Zhongdu; ST36, Zusanli; ST43, Xiangu the right side | BOLD fMRI | SPM2 | Talairach |
| Dougherty et al. (2008) | Sham - True acupuncture | 3 male and 3 females in each group with the age (mean ± S.D.) of 28 ± 7 years for the acupuncture group and 36 ± 10 years for the placebo acupuncture group | Manual acupuncture at Large Intestine 4 (LI 4) on the right han | [11C]diprenorphine | SPM2 | MNI |
| Wu et al. (1999) | Sham - True acupuncture | 9 (6 women, 20-35 years) | Manual acupuncture at ST36 amd LI4 | BOLD fMRI |  | Talairach |
| Wu et al. (2002) | Sham - True acupuncture | 15 healthy volunteers (age 20–30 years; 10 males, 5 females), | Electroacupuncture at GB34, “Yanglinquan” | BOLD fMRI | SPM99 | Talairach |
| Kong et al. (2002) | Block-designed study | 11 righthanded normal, healthy volunteer adults, 6 male and 5 female, ages 21–64 (32 6 16.1) | Manual acupuncture and electroacupuncture at acupoint LI4 on the left hand only | BOLD fMRI | AFNI version 2.31b and SPM | Talairach |
| MacPherson et al. (2008) | Block-designed study | 8 males (mean age 33 years, range 18–46, S.D. = 11.9) and 9 females (mean age 39 years, range 20–54, S.D. = 10.2), with an average age of 36 years | Manual acupuncture at Hegu (LI-4) on the right hand | BOLD fMRI | FSL | MNI |
| Li et al. (2003) | Block-designed study | 10 healthy subjects | Manual acupuncture and electroacupuncture at (bladder (BL) 60, BL65, BL66, and BL67) [5,11] located in the  lateral aspect of right foot | BOLD fMRI | SPM | Talairach |
| Li et al. (2006) | Pre - post acupuncture | 18 (mean age standard deviation [SD]: 26.6 3.2 years; range  23–33 years; 11 male, 7 female) | Manual acupuncture at acupoint LI4 (Hegu right thenar muscle. | BOLD fMRI | SPM2 | Talairach |
| Li et al. (2016) | Sham - True acupuncture | 12 healthy right-handed male participants (range: 23–26 years; mean: 24.8 years) | Manual acupuncture at KI 2 | BOLD fMRI | SPM12 | MNI |
| Wik et al. (2016) | Sham - True acupuncture | 12 male and 12 female  right-handed volunteers with a mean age of 23.1 2.2  years (range, 21e28 years) | Manual acupuncture at SJ 5 acupoint | BOLD fMRI | SPM2 | Talairach |
| Yeo et al. (2016) | Sham - True acupuncture | 19 healthy  participants (9 male, 10 female) 48.1±3.38 | Manual acupuncture at GB34 on the right side | BOLD fMRI | SPM5 | Talairach |
| Lv et al. (2016) | Pre - post acupuncture | 21 (18 to  27 (mean age: 21.9 years) | Laser acupuncture at HI11 and CV1 GV26 (renzhong), CV24 (chengjiang), ST6 (jiache), BL62 (shenmai), SP1 (yinbai), and LU11 (shaoshang), PC7 (daling), PC8 (laogong) | BOLD fMRI | SPM8 | MNI |
| Chung et al. (2019) | Pre - post acupuncture | 10 healthy participants | Manual acupuncture at MS5, left MS6, and left MS7 | BOLD fMRI | SPM12 and REST | MNI |
| Wei et al. (2020) | Block-designed study | 16 right-handed acupuncture-naive healthy  volunteers (8 females and 8 males) aged 21–30 years (mean  24.1 years) | Manual and Ultrasound stimulation ST 36. | BOLD fMRI | SPM12 | MNI |
| Xu et al. (2022) | Pre - post acupuncture | 20 healthy female subjects | Bilateral uterine points  (EX-CA1) by electroacupuncture | BOLD fMRI | DPABI | MNI |
| Shan et al. (2018) | Sham - True acupuncture | 14 healthy subjects (66.1 years) | Manual acupuncture at Siguan | BOLD fMRI | SPM12 | MNI |
| Yoon et al. (2023) | Pre - post acupuncture | 25 healthy participants (12 females, 24.2 ± 0.81 years) | Manual acupuncture at left ST36 | BOLD fMRI | SPM12 and AFNI | MNI |
| **Patients with various of diseases** | | | | | | |
| Chen et al. (2020) | Pre - post acupuncture | 10 patients with ischemic stroke (7 men, 57.70 ± 7.69 years) | Manual acupuncture at Quchi and Zusanli (LI 11 and ST 36) on the right side for 15 mins | BOLD fMRI | DPARSF and REST | MNI |
| Fang et al. (2012) | Pre - post acupuncture | 6 patients with ischemic stroke (3 men, ages 50–75 years) | Electroacupuncture at Baihui (GV 20) and right Qubin (GB 7) for 20 mins | ^18^F-FDG PET/CT | SPM99 | MNI |
| Zhao et al. (2014) | Sham - True acupuncture | 40 migraineurs without aura (14 men, ages 35.08 ± 10.34 years ) | Manual acupuncture at acupuncture at bilateral Waiguan (TE5), Fengchi (GB 20), Yanglingquan (GB 34), and Qiuxu (GB 40); and sham acupuncture on bilateral Erheliao (TE 22), Daling (PC 7), Guangming (GB 37), and Taibai (SP 3) | BOLD fMRI | SPM5 | Talairach |
| Yang et al. (2012); Yang et al. (2014) | Sham - True acupuncture | 30 patients with acute  migraine without aura (12 men, ages 33.28 ± 8.03 years) | Electroacupuncture at Shaoyang meridians, Luxi (TE 19), Sanyangluo (TE 8), and Xiyangguan (GB 33) for 30 mins. | ^18^F-FDG PET/CT | SPM2 | MNI |
| Li et al. (2017) | Sham - True acupuncture | 62 patients with migraine without aura (14 men, mean age 21.29 years) | Manual acupuncture for Group 1: Yanglingquan (GB 34), Qiuxu (GB 40) and Waiguan (TE 5). Group 2: Xiyangguan (GB 33), Diwuhui (GB 42) and Sanyangluo (TE 8). Group 3: Zusanli (ST 36), Chongyang (ST 42) and Pianli (LI 6). Sham acupoints at three adjacent non‐acupoints. | BOLD fMRI | SPM12 and DPARSF | MNI |
| Liu et al. (2014) | Pre - post acupuncture | 15 female depressive patients (ages 41.03 ± 11.36 years ) | Manual acupuncture at Zhongwan (CV 12), Xiawan (CV 10), Qihai (CV 6), Guanyuan (CV 4), Shangqu (KI 17), Huaroumen (ST 24), and Qipang (extra-point). | BOLD fMRI | SPM8 and DPARSF | MNI |
| Wang et al. (2017) | Sham - True acupuncture | 46 female major depressive patients (44.14 ± 8.78) | Manual acupuncture at Zhongwan (CV 12), Xiawan (CV 10), Qihai (CV 6), Guanyuan (CV 4), Shangqu (KI 17), Huaroumen (ST 24), and Qipang (extra-point). | BOLD fMRI | SPM8 | MNI |
| Pang et al. (2018) | Pre - post acupuncture | 20 patients with premenstrual syndrome (ages 21.85 ± 1.72) years) | Electroacupuncture at Sanyinjiao (SP 6); | BOLD fMRI | SPM8 | MNI |
| Shan et al. (2018) | Sham - True acupuncture | 21 Alzheimer’s disease patients (9 men, mean age 66.9 years), 14 mild cognitive impairment (6 men, mean age 66.4 years), and 14 healthy people (6 men, mean age 66.1 years) | Manual acupuncture at bilateral Taichong (LR 3), bilateral Hegu (LI 4), bilateral Taichong (LR 3) or Hegu (LI 4). | BOLD fMRI | SPM12 | MNI |
| Z. Li et al. (2018) | Sham - True acupuncture | 41 patients with Parkinson’s disease (24 men, ages 63.60 ± 6.24 years) | Manual acupuncture at Baihui (GV20), Fengchi (GB20), and Chorea-Tremor Controlled Zone for 12 weeks | BOLD fMRI | DPABI | MNI |
| Yeo et al. (2018) | Pre - post acupuncture | 10 idiopathic patients with Parkinson’s disease (5 men, ages 56.9 ± 9.49 years) | Electroacupuncture at right Yanglingquan (GB 34), right Taechung (LR 3), Quchi (LI 11), Zusanli (ST 36), Fengchi (GB 20), Sanyinjiao (SP 6), Hegu (LI 4), and Yanglingquan (GB 34). For 8 weeks, two times a week. | BOLD fMRI | SPM5 | MNI |
| (Yan et al., 2020; Zhang et al., 2018) | Pre - post acupuncture | Chronic shoulder pain: 12 contralateral (6 men, ages 53.33 ± 5.26 years) 8 ipsilateral (4men, ages 54.13 ± 7.45 years) | Manual acupuncture at contralateral or ipsilateral Tiaokou (ST 38). | BOLD fMRI | SPM12 and DPARSF | MNI |
| J. Zhang et al. (2021) | Sham - True acupuncture | 15 patients with hypertension in Group 1 (5 men), 14 patients in Group 2 (5men) | Manual acupuncture for Group 1: Taichong (LR 3) +Taixi (KI 3); Group2: Taichong (LR 3) + sham | BOLD fMRI | REST 1.8 | MNI |
| Liu et al. (2020) | Pre - post acupuncture | 12 sciatica patients (6 men, ages 61.42 ± 14.84 years) | Manual acupuncture at Shenshu (BL 23), Huantiao (GB 30), Weizhong (BL 40), Yanglingquan (GB 34), Kunlun (BL 60), and Xuanzhong (GB 39). The eighteen acupoints were Shenshu (BL 23), Dachangshu (BL 25), Xiaochangshu (BL 27), Huantiao (GB 30), Yinmen (BL 37), Zhibian (BL 54), Chengfu (BL 36), Fengshi (GB 31), Weizhong (BL 40), Zusanli (ST 36), Yanglingquan (GB 34), Yinlingquan (SP 9), Feiyang (BL 58), Sanyinjiao (SP 6), Xuanzhong (GB 39), Kunlun (BL 60), Taixi (KI 3), and Shenmai (BL 62) | BOLD fMRI | DPARSF | MNI |
| Bao et al. (2016) | Pre - post acupuncture | 18 patients with emissive Crohn’s Disease (13 men, ages 31.61 ± 5.00 years) | Electroacupuncture at the Tianshu (ST 25) and Qihai (CV 6) at the left-hand side, as well as the Tianshu (ST 25) and Zhongwan (CV 12) at the right-hand side. | BOLD fMRI | SPM8 | MNI |
| Shi et al. (2021) | Pre - post acupuncture | 30 primary insomnia (12 women, ages 48.94 ± 8.63 years) | Electroacupuncture treatment on HT-7 for 5-week | BOLD fMRI | DPARSF | MNI |
| Chae, Lee, Kim, Kim, et al. (2009) | Block design study | 10 (six men, four women; age range: 45–66 years; disease duration 3.0 6 2.0 years; | Manual acupuncture GB34 | BOLD fMRI | SPM5 | Talairach |
| Zhang et al. (2019) | Pre - post acupuncture | 20 | Manual acupuncture at LR3 and KI3 | BOLD fMRI | SPM8 and REST | MNI |
| Wang et al. (2020) | Pre - post acupuncture | S-Acu group(43, 55.42 ± 9.23) M-Acu group (43, 51.45 ± 12.10) | Manual acupuncture at Shenmen (HT-7), Sanyinjiao (SP-6), Baihui (GV-20) | BOLD fMRI | DPARSF | MNI |
| H. Liu et al. (2021) | Sham - True acupuncture | 13 patients with stroke | Manual acupuncture at the midline of vertex (MS5), the left anterior oblique line of vertextemporal (MS6) and the left posterior oblique line of vertex-temporal (MS7). | BOLD fMRI | SPM12 | MNI |
| S. Liu et al. (2021) | Pre - post acupuncture | 37 patients with migraine without Aura | Manual acupuncture at Baihui (DU20), Taiyang (EX-HN5), bilateral Fengchi (GB20), Shuaigu (GB8), Xuanlu (GB5), Toulinqi (GB15), Hegu (LI4), and Taichong (LR3) | BOLD fMRI | SPM12 | MNI |
| Wei et al. (2021) | Pre - post acupuncture | 20 (10males)26.45 ± 1.82 (22–30) | Electroacupuncture was performed at GV20 acupoint | BOLD fMRI | DPABI | MNI |
| Y. Zhang et al. (2021) | Sham - True acupuncture | 24 patients with migraine | Manual acupuncture at GB20 (Fengchi), GB8 (Shuaigu), PC6 (Neiguan), SP6 (Sanyinjiao), and LR3 (Taichong) | BOLD fMRI | DPABI | Talairach |
| Duan et al. (2021) | Pre - post acupuncture | 20 patients with premenstrual syndrome (21.85 ± 1.72 years) | Electroacupuncture the left leg at acupoint SP6 | BOLD fMRI | DPARSF | MNI |
| Lan et al. (2022) | Pre - post acupuncture | 29 patients with chronic stable angina pectoris | Manual acupuncture at Group A (acupoints on the meridian directly related to the Heart): bilateral Neiguan (PC6) and bilateral Tongli (HT5); Group B (acupoints on the meridian indirectly related to the Heart): bilateral Yangxi (LI5) and bilateral Pianli (LI6). | BOLD fMRI | DPABI | MNI |
| Li et al. (2022) | Pre - post acupuncture | 24 volunteers (14 males) with anxiety disorders | Manual acupuncture at left Neiguan for 15 minutes | BOLD fMRI | DPARSF 2.3 | MNI |
| Liu et al. (2022) | Pre - post acupuncture | 20 participants (24.70 ± 2.11) with PDM | Manual acupuncture at bilateral SP6 | BOLD fMRI | SPM12 and BRANT toolkit | MNI |
| Teng et al. (2022) | Pre - post acupuncture | 115 FD patients | Study1: Manual acupuncture at ST36 combined with CV12 ; Study 2: ST36 with Deqi and without Deqi | BOLD fMRI | SPM12 and DPARSF 4.5 | MNI |
| Zhou et al. (2024) | Pre - post acupuncture | 30 patients (19 males,63.17 ± 10.31 years) | Manual acupuncture at Dazhui (GV14), Zhiyang (GV9) and Mingmen (GV4) | BOLD fMRI | DPABI | MNI |
| Wang et al. (2023) | Sham - True acupuncture | 36 patients with anxiety disorders (aged 20 to 35 years) | Manual acupuncture at LU10 for 20 minutes | BOLD fMRI | NeuroScholar cloud platform | MNI |
| Yang et al. (2023) | Pre - post acupuncture | 25 CSVDCI patients (13 males, 61.9 ± 4.56 years) | Manual acupuncture at Shenting and Baihui. | BOLD fMRI | DPARSF | MNI |
| Yang et al. (2024) | Pre - post acupuncture | 50 patients (28.36 ± 3.61) | Electroacupuncture at Baihui (DU20), Shuaigu (GB8), Xuanlu (GB5), and Touwei (ST8) | BOLD fMRI | SPM 12 | MNI |

References:

An, Y. S., Moon, S. K., Min, I. K., & Kim, D. Y. (2009). Changes in regional cerebral blood flow and glucose metabolism following electroacupuncture at LI 4 and LI 11 in normal volunteers. *J Altern Complement Med*, *15*(10), 1075-1081. <https://doi.org/10.1089/acm.2009.0257>

Bao, C., Liu, P., Liu, H., Jin, X., Calhoun, V. D., Wu, L., Shi, Y., Zhang, J., Zeng, X., & Ma, L. (2016). Different brain responses to electro-acupuncture and moxibustion treatment in patients with Crohn’s disease. *Scientific Reports*, *6*(1), 1-12.

Biella, G., Sotgiu, M. L., Pellegata, G., Paulesu, E., Castiglioni, I., & Fazio, F. (2001). Acupuncture produces central activations in pain regions. *Neuroimage*, *14*(1 Pt 1), 60-66. <https://doi.org/10.1006/nimg.2001.0798>

Cao, J., Tu, Y., Wilson, G., Orr, S. P., & Kong, J. (2020). Characterizing the analgesic effects of real and imagined acupuncture using functional and structure MRI. *Neuroimage*, *221*, 117176.

Chae, Y., Lee, H., Kim, H., Kim, C. H., Chang, D. I., Kim, K. M., & Park, H. J. (2009). Parsing brain activity associated with acupuncture treatment in Parkinson's diseases. *Mov Disord*, *24*(12), 1794-1802. <https://doi.org/10.1002/mds.22673>

Chae, Y., Lee, H., Kim, H., Sohn, H., Park, J. H., & Park, H. J. (2009). The neural substrates of verum acupuncture compared to non-penetrating placebo needle: an fMRI study. *Neurosci Lett*, *450*(2), 80-84. <https://doi.org/10.1016/j.neulet.2008.11.048>

Chen, S. Q., Cai, D. C., Chen, J. X., Yang, H., & Liu, L. S. (2020). Altered Brain Regional Homogeneity Following Contralateral Acupuncture at Quchi (LI 11) and Zusanli (ST 36) in Ischemic Stroke Patients with Left Hemiplegia: An fMRI Study. *Chin J Integr Med*, *26*(1), 20-25. <https://doi.org/10.1007/s11655-019-3079-6>

Chung, W. Y., Liu, S. Y., Gao, J. C., Jiang, Y. J., Zhang, J., Qu, S. S., Zhang, J. P., Tan, X. L., Chen, J. Q., & Wang, S. X. (2019). Modulatory effect of International Standard Scalp Acupuncture on brain activation in the elderly as revealed by resting-state fMRI. *Neural Regen Res*, *14*(12), 2126-2131. <https://doi.org/10.4103/1673-5374.262590>

Dougherty, D. D., Kong, J., Webb, M., Bonab, A. A., Fischman, A. J., & Gollub, R. L. (2008). A combined [11C]diprenorphine PET study and fMRI study of acupuncture analgesia. *Behav Brain Res*, *193*(1), 63-68. <https://doi.org/10.1016/j.bbr.2008.04.020>

Duan, G., Chen, Y., Pang, Y., Feng, Z., Liao, H., Liu, H., Zou, Z., Li, M., Tao, J., He, X., Li, S., Liu, P., & Deng, D. (2021). Altered fractional amplitude of low-frequency fluctuation in women with premenstrual syndrome via acupuncture at Sanyinjiao (SP6). *Ann Gen Psychiatry*, *20*(1), 29. <https://doi.org/10.1186/s12991-021-00349-z>

Fang, J., Jin, Z., Wang, Y., Li, K., Kong, J., Nixon, E. E., Zeng, Y., Ren, Y., Tong, H., Wang, Y., Wang, P., & Hui, K. K. (2009). The salient characteristics of the central effects of acupuncture needling: limbic-paralimbic-neocortical network modulation. *Human Brain Mapping*, *30*(4), 1196-1206. <https://doi.org/10.1002/hbm.20583>

Fang, Z., Ning, J., Xiong, C., & Shulin, Y. (2012). Effects of electroacupuncture at head points on the function of cerebral motor areas in stroke patients: a PET study. *Evidence-Based Complementary and Alternative Medicine*, *2012*.

He, L., Chen, G., Zheng, R., Hu, Y., Chen, X., & Ruan, J. (2020). Heterogeneous Acupuncture Effects of Taixi (KI3) on Functional Connectivity in Healthy Youth and Elder: A Functional MRI Study Using Regional Homogeneity and Large-Scale Functional Connectivity Analysis. *Neural Plast*, *2020*, 8884318. <https://doi.org/10.1155/2020/8884318>

Hui, K. K., Marina, O., Claunch, J. D., Nixon, E. E., Fang, J., Liu, J., Li, M., Napadow, V., Vangel, M., Makris, N., Chan, S. T., Kwong, K. K., & Rosen, B. R. (2009). Acupuncture mobilizes the brain's default mode and its anti-correlated network in healthy subjects. *Brain Res*, *1287*, 84-103. <https://doi.org/10.1016/j.brainres.2009.06.061>

Jiang, Y., Wang, H., Liu, Z., Dong, Y., Dong, Y., Xiang, X., Bai, L., Tian, J., Wu, L., Han, J., & Cui, C. (2013). Manipulation of and sustained effects on the human brain induced by different modalities of acupuncture: an fMRI study. *PLoS One*, *8*(6), e66815. <https://doi.org/10.1371/journal.pone.0066815>

Jin, L., Sun, J., Xu, Z., Yang, X., Liu, P., & Qin, W. (2018). Intersubject synchronisation analysis of brain activity associated with the instant effects of acupuncture: an fMRI study. *Acupunct Med*, *36*(1), 14-20. <https://doi.org/10.1136/acupmed-2016-011327>

Kong, J., Gollub, R. L., Webb, J. M., Kong, J. T., Vangel, M. G., & Kwong, K. (2007). Test-retest study of fMRI signal change evoked by electroacupuncture stimulation. *Neuroimage*, *34*(3), 1171-1181. <https://doi.org/10.1016/j.neuroimage.2006.10.019>

Kong, J., Ma, L., Gollub, R. L., Wei, J., Yang, X., Li, D., Weng, X., Jia, F., Wang, C., Li, F., Li, R., & Zhuang, D. (2002). A pilot study of functional magnetic resonance imaging of the brain during manual and electroacupuncture stimulation of acupuncture point (LI-4 Hegu) in normal subjects reveals differential brain activation between methods. *J Altern Complement Med*, *8*(4), 411-419. <https://doi.org/10.1089/107555302760253603>

Lai, X., Zhang, G., Huang, Y., Tang, C., Yang, J., Wang, S., & Zhou, S. F. (2009). A cerebral functional imaging study by positron emission tomography in healthy volunteers receiving true or sham acupuncture needling. *Neurosci Lett*, *452*(2), 194-199. <https://doi.org/10.1016/j.neulet.2009.01.052>

Lan, L., Yin, T., Tian, Z., Lan, Y., Sun, R., Li, Z., Jing, M., Wen, Q., Li, S., Liang, F., & Zeng, F. (2022). Acupuncture Modulates the Spontaneous Activity and Functional Connectivity of Calcarine in Patients With Chronic Stable Angina Pectoris. *Front Mol Neurosci*, *15*, 842674. <https://doi.org/10.3389/fnmol.2022.842674>

Li, A., Li, X. L., Zhang, F., Yue, J. H., Yuan, C. S., Li, K., & Zhang, Q. H. (2016). A functional magnetic resonance imaging study of the neuronal specificity of an acupoint: acupuncture at Rangu (KI 2) and its sham point. *Intern Med J*, *46*(8), 973-977. <https://doi.org/10.1111/imj.13154>

Li, A., Wang, Y. H., Zhang, F., Wang, F., Zeng, X. X., Yue, J. H., Li, X. L., & Zhang, Q. H. (2018). Acupuncture for gender differences and similarities in cerebral activity of health volunteers: A pilot fMRI study. *Medicine (Baltimore)*, *97*(50), e13655. <https://doi.org/10.1097/MD.0000000000013655>

Li, C., Wang, Y., Li, B., & Su, S. (2022). Effects of acupuncture at neiguan in neural activity of related brain regions: a resting-state fMRI study in anxiety. *Neuropsychiatric Disease and Treatment*, *18*, 1375.

Li, G., Cheung, R. T., Ma, Q. Y., & Yang, E. S. (2003). Visual cortical activations on fMRI upon stimulation of the vision-implicated acupoints. *Neuroreport*, *14*(5), 669-673. <https://doi.org/10.1097/00001756-200304150-00002>

Li, K., Shan, B., Xu, J., Liu, H., Wang, W., Zhi, L., Li, K., Yan, B., & Tang, X. (2006). Changes in FMRI in the human brain related to different durations of manual acupuncture needling. *J Altern Complement Med*, *12*(7), 615-623. <https://doi.org/10.1089/acm.2006.12.615>

Li, L., Liu, H., Li, Y. Z., Xu, J. Y., Shan, B. C., Gong, D., Li, K. C., & Tang, X. W. (2008). The human brain response to acupuncture on same-meridian acupoints: evidence from an fMRI study. *J Altern Complement Med*, *14*(6), 673-678. <https://doi.org/10.1089/acm.2008.0036>

Li, L. L., Liu, X. W., Wu, F., Tong, D. C., Ye, L. P., Tao, H. X., Liu, P., Qiu, Y. H., & Yang, W. Z. (2018). Electroacupuncture Stimulation of Language-Implicated Acupoint Tongli (HT 5) in Healthy Subjects: An fMRI Evaluation Study. *Chin J Integr Med*, *24*(11), 822-829. <https://doi.org/10.1007/s11655-017-2924-8>

Li, Z., Chen, J., Cheng, J., Huang, S., Hu, Y., Wu, Y., Li, G., Liu, B., Liu, X., Guo, W., Huang, S., Zhou, M., Chen, X., Xiao, Y., Chen, C., Chen, J., Luo, X., & Xu, P. (2018). Acupuncture Modulates the Cerebello-Thalamo-Cortical Circuit and Cognitive Brain Regions in Patients of Parkinson's Disease With Tremor. *Front Aging Neurosci*, *10*, 206. <https://doi.org/10.3389/fnagi.2018.00206>

Li, Z., Zeng, F., Yin, T., Lan, L., Makris, N., Jorgenson, K., Guo, T., Wu, F., Gao, Y., Dong, M., Liu, M., Yang, J., Li, Y., Gong, Q., Liang, F., & Kong, J. (2017). Acupuncture modulates the abnormal brainstem activity in migraine without aura patients. *Neuroimage Clin*, *15*, 367-375. <https://doi.org/10.1016/j.nicl.2017.05.013>

Liu, C. H., Yeh, T. C., Kung, Y. Y., Tseng, H. P., Yang, C. J., Hong, T. Y., Cheng, C. M., Yang, J. L., Wu, T. P., Hsieh, J. C., & Chen, F. P. (2020). Changes in resting-state functional connectivity in nonacute sciatica with acupuncture modulation: A preliminary study. *Brain Behav*, *10*(2), e01494. <https://doi.org/10.1002/brb3.1494>

Liu, H., Jiang, Y., Wang, N., Yan, H., Chen, L., Gao, J., Zhang, J., Qu, S., Liu, S., Liu, G., Huang, Y., & Chen, J. (2021). Scalp acupuncture enhances local brain regions functional activities and functional connections between cerebral hemispheres in acute ischemic stroke patients. *Anat Rec (Hoboken)*, *304*(11), 2538-2551. <https://doi.org/10.1002/ar.24746>

Liu, J., Liu, B., Nie, G., Wang, B., Feng, X., & Wang, X. (2014). Regional homogeneity change in female depressive patients after abdominal acupuncture treatment. 2014 IEEE International Conference on Bioinformatics and Biomedicine (BIBM),

Liu, L.-Y., Li, X., Tian, Z.-L., Zhang, Q., Shen, Z.-F., Wei, W., Guo, X.-L., Chen, L., Su, M.-H., & Yang, L. (2022). Acupuncture modulates the frequency-specific functional connectivity density in primary dysmenorrhea. *Frontiers in Neuroscience*, *16*, 917721.

Liu, L., Chen, S., Zeng, D., Li, H., Shi, C., & Zhang, L. (2018). Cerebral activation effects of acupuncture at Yanglinquan (GB34) point acquired using resting-state fMRI. *Computerized Medical Imaging and Graphics*, *67*, 55-58.

Liu, L., Wu, Y., Zheng, J., Lai, X., Zeng, D., Li, H., Shi, C., Yang, M., & Liang, C. (2016). Cerebral activation effects of acupuncture using Zusanli (ST36) and Yanglingquan (GB34) points based on Regional Homogeneity indices: A resting-state fMRI study. *J Xray Sci Technol*, *24*(2), 297-308. <https://doi.org/10.3233/XST-160557>

Liu, S., Luo, S., Yan, T., Ma, W., Wei, X., Chen, Y., Zhan, S., & Wang, B. (2021). Differential Modulating Effect of Acupuncture in Patients With Migraine Without Aura: A Resting Functional Magnetic Resonance Study. *Front Neurol*, *12*, 680896. <https://doi.org/10.3389/fneur.2021.680896>

Long, X., Huang, W., Napadow, V., Liang, F., Pleger, B., Villringer, A., Witt, C. M., Nierhaus, T., & Pach, D. (2016). Sustained Effects of Acupuncture Stimulation Investigated with Centrality Mapping Analysis. *Frontiers in Human Neuroscience*, *10*, 510. <https://doi.org/10.3389/fnhum.2016.00510>

Lv, J., Shi, C., Deng, Y., Lou, W., Hu, J., Shi, L., Luo, L., & Wang, D. (2016). The brain effects of laser acupuncture at thirteen ghost acupoints in healthy individuals: A resting-state functional MRI investigation. *Comput Med Imaging Graph*, *54*, 48-54. <https://doi.org/10.1016/j.compmedimag.2016.08.003>

MacPherson, H., Green, G., Nevado, A., Lythgoe, M. F., Lewith, G., Devlin, R., Haselfoot, R., & Asghar, A. U. (2008). Brain imaging of acupuncture: comparing superficial with deep needling. *Neurosci Lett*, *434*(1), 144-149. <https://doi.org/10.1016/j.neulet.2008.01.058>

Nierhaus, T., Pach, D., Huang, W., Long, X., Napadow, V., Roll, S., Liang, F., Pleger, B., Villringer, A., & Witt, C. M. (2015). Differential cerebral response to somatosensory stimulation of an acupuncture point vs. two non-acupuncture points measured with EEG and fMRI. *Frontiers in Human Neuroscience*, *9*, 74. <https://doi.org/10.3389/fnhum.2015.00074>

Pang, Y., Liu, H., Duan, G., Liao, H., Liu, Y., Feng, Z., Tao, J., Zou, Z., Du, G., & Wan, R. (2018). Altered brain regional homogeneity following electro-acupuncture stimulation at sanyinjiao (SP6) in women with premenstrual syndrome. *Frontiers in Human Neuroscience*, *12*, 104.

Quah-Smith, I., Williams, M. A., Lundeberg, T., Suo, C., & Sachdev, P. (2013). Differential brain effects of laser and needle acupuncture at LR8 using functional MRI. *Acupunct Med*, *31*(3), 282-289. <https://doi.org/10.1136/acupmed-2012-010297>

Shan, Y., Wang, J.-J., Wang, Z.-Q., Zhao, Z.-L., Zhang, M., Xu, J.-Y., Han, Y., Li, K.-C., & Lu, J. (2018). Neuronal specificity of acupuncture in Alzheimer’s disease and mild cognitive impairment patients: a functional MRI study. *Evidence-Based Complementary and Alternative Medicine*, *2018*.

Shi, X. H., Wang, Y. K., Li, T., Liu, H. Y., Wang, X. T., Wang, Z. H., Mang, J., & Xu, Z. X. (2021). Gender-related difference in altered fractional amplitude of low-frequency fluctuations after electroacupuncture on primary insomnia patients: A resting-state fMRI study. *Brain Behav*, *11*(1), e01927. <https://doi.org/10.1002/brb3.1927>

Teng, Y., Yin, T., Yang, Y., Sun, R., Tian, Z., Ma, P., He, Z., Qu, Y., Huang, L., & Chen, Y. (2022). The role of medial prefrontal cortex in acupuncture treatment for functional dyspepsia. *Frontiers in Neuroscience*, *16*, 801899.

Wang, F., Yang, T., Li, X., Liu, X., Cao, D., Wang, D., Yang, Y., Li, C., Qu, Y., Zhao, X., Sun, Z., & Asakawa, T. (2021). Cerebral areas affected by unilateral acupuncture on SP3 in healthy volunteers: An explorative resting-state fMRI study. *Brain Behav*, *11*(4), e02057. <https://doi.org/10.1002/brb3.2057>

Wang, W., Liu, L., Zhi, X., Huang, J. B., Liu, D. X., Wang, H., Kong, X. Q., & Xu, H. B. (2007). Study on the regulatory effect of electro-acupuncture on hegu point (LI4) in cerebral response with functional magnetic resonance imaging. *Chin J Integr Med*, *13*(1), 10-16. <https://doi.org/10.1007/s11655-007-0010-3>

Wang, Y., Li, C., & Qi, X. (2023). The effect of acupuncture at the Yuji point on resting-state brain function in anxiety. *Medicine*, *102*(8), e33094.

Wang, Y. K., Li, T., Ha, L. J., Lv, Z. W., Wang, F. C., Wang, Z. H., Mang, J., & Xu, Z. X. (2020). Effectiveness and cerebral responses of multi-points acupuncture for primary insomnia: a preliminary randomized clinical trial and fMRI study. *BMC Complement Med Ther*, *20*(1), 254. <https://doi.org/10.1186/s12906-020-02969-6>

Wang, Z., Wang, X., Liu, J., Chen, J., Liu, X., Nie, G., Jorgenson, K., Sohn, K. C., Huang, R., Liu, M., Liu, B., & Kong, J. (2017). Acupuncture treatment modulates the corticostriatal reward circuitry in major depressive disorder. *J Psychiatr Res*, *84*, 18-26. <https://doi.org/10.1016/j.jpsychires.2016.09.014>

Wei, X. Y., Chen, H., Guo, C., Tan, W. L., & Zhan, S. H. (2021). The Instant and Sustained Effect of Electroacupuncture in Postgraduate Students with Depression: An fMRI Study. *Neuropsychiatr Dis Treat*, *17*, 873-883. <https://doi.org/10.2147/ndt.S307083>

Wei, Y., Mei, L., Long, X., Wang, X., Diao, Y., Nguchu, B. A., Hu, S., Wang, Y., Yu, H., & Qiu, B. (2020). Functional MRI Investigation of Ultrasound Stimulation at ST 36. *Evid Based Complement Alternat Med*, *2020*, 6794013. <https://doi.org/10.1155/2020/6794013>

Wik, G., Huang, Y., Zeng, T., Qu, S., Zheng, Y., Zhang, J., Lai, X., Tang, C., & Shan, B. (2016). Waiguan Stimulation May Kindle Anticorrelated Brain Networks: Functional Magnetic Resonance Imaging Data Revisited. *J Acupunct Meridian Stud*, *9*(1), 22-25. <https://doi.org/10.1016/j.jams.2015.11.035>

Wu, M. T., Hsieh, J. C., Xiong, J., Yang, C. F., Pan, H. B., Chen, Y. C., Tsai, G., Rosen, B. R., & Kwong, K. K. (1999). Central nervous pathway for acupuncture stimulation: localization of processing with functional MR imaging of the brain--preliminary experience. *Radiology*, *212*(1), 133-141. <https://doi.org/10.1148/radiology.212.1.r99jl04133>

Wu, M. T., Sheen, J. M., Chuang, K. H., Yang, P., Chin, S. L., Tsai, C. Y., Chen, C. J., Liao, J. R., Lai, P. H., Chu, K. A., Pan, H. B., & Yang, C. F. (2002). Neuronal specificity of acupuncture response: a fMRI study with electroacupuncture. *Neuroimage*, *16*(4), 1028-1037. <https://doi.org/10.1006/nimg.2002.1145>

Xu, C., Yu, X., Yin, L., Li, X., Zhang, W., Li, F., & Bai, T. (2022). Functional Magnetic Resonance Imaging Study of Electroacupuncture Stimulating Uterine Acupoints. *Comput Math Methods Med*, *2022*, 4295985. <https://doi.org/10.1155/2022/4295985>

Yan, C. Q., Huo, J. W., Wang, X., Zhou, P., Zhang, Y. N., Li, J. L., Kim, M., Shao, J. K., Hu, S. Q., Wang, L. Q., & Liu, C. Z. (2020). Different Degree Centrality Changes in the Brain after Acupuncture on Contralateral or Ipsilateral Acupoint in Patients with Chronic Shoulder Pain: A Resting-State fMRI Study. *Neural Plast*, *2020*, 5701042. <https://doi.org/10.1155/2020/5701042>

Yang, C. H., Choi, S. H., Kim, J. S., Ryu, Y. H., Lim, Y. J., Kim, M. S., Sohn, J. W., Oh, S. S., Kim, C., & Lee, M. Y. (2017). The Effects of Acupuncture Stimulation for Brain Activation and Alcohol Abstinence Self-Efficacy: Functional MRI Study. *Evid Based Complement Alternat Med*, *2017*, 2850124. <https://doi.org/10.1155/2017/2850124>

Yang, J., Zeng, F., Feng, Y., Fang, L., Qin, W., Liu, X., Song, W., Xie, H., Chen, J., & Liang, F. (2012). A PET-CT study on the specificity of acupoints through acupuncture treatment in migraine patients. *BMC Complement Altern Med*, *12*, 123. <https://doi.org/10.1186/1472-6882-12-123>

Yang, M., Yang, J., Zeng, F., Liu, P., Lai, Z., Deng, S., Fang, L., Song, W., Xie, H., & Liang, F. (2014). Electroacupuncture stimulation at sub-specific acupoint and non-acupoint induced distinct brain glucose metabolism change in migraineurs: a PET-CT study. *J Transl Med*, *12*, 351. <https://doi.org/10.1186/s12967-014-0351-6>

Yang, N., Chen, S., Liu, S., Ling, S., & Chen, L. (2023). Increased low frequency fluctuation in the brain after acupuncture treatment in CSVDCI patients: A randomized control trial study. *Frontiers in Neuroscience*, *17*, 1125418.

Yang, Y.-C., Wei, X.-Y., Zhang, Y.-Y., Xu, C.-Y., Cheng, J.-M., Gong, Z.-G., Chen, H., Huang, Y.-W., Yuan, J., & Xu, H.-H. (2024). Modulation of temporal and occipital cortex by acupuncture in non-menstrual MWoA patients: a rest BOLD fMRI study. *BMC Complementary Medicine and Therapies*, *24*(1), 43.

Yeo, S., Rosen, B., Bosch, P., Noort, M. V., & Lim, S. (2016). Gender differences in the neural response to acupuncture: clinical implications. *Acupunct Med*, *34*(5), 364-372. <https://doi.org/10.1136/acupmed-2015-011025>

Yeo, S., van den Noort, M., Bosch, P., & Lim, S. (2018). A study of the effects of 8-week acupuncture treatment on patients with Parkinson's disease. *Medicine (Baltimore)*, *97*(50), e13434. <https://doi.org/10.1097/MD.0000000000013434>

Yoo, S. S., Kerr, C. E., Park, M., Im, D. M., Blinder, R. A., Park, H., & Kaptchuk, T. J. (2007). Neural activities in human somatosensory cortical areas evoked by acupuncture stimulation. *Complement Ther Med*, *15*(4), 247-254. <https://doi.org/10.1016/j.ctim.2007.01.010>

Yoo, S. S., Teh, E. K., Blinder, R. A., & Jolesz, F. A. (2004). Modulation of cerebellar activities by acupuncture stimulation: evidence from fMRI study. *Neuroimage*, *22*(2), 932-940. <https://doi.org/10.1016/j.neuroimage.2004.02.017>

Yoon, D.-E., Lee, S., Kim, J., Kim, K., Park, H.-J., Napadow, V., Lee, I.-S., & Chae, Y. (2023). Graded brain fMRI response to somatic and visual acupuncture stimulation. *Cerebral Cortex*, *33*(23), 11269-11278.

Zhang, J., Cai, X., Wang, Y., Zheng, Y., Qu, S., Zhang, Z., Yao, Z., Chen, G., Tang, C., & Huang, Y. (2019). Different Brain Activation after Acupuncture at Combined Acupoints and Single Acupoint in Hypertension Patients: An Rs-fMRI Study Based on ReHo Analysis. *Evid Based Complement Alternat Med*, *2019*, 5262896. <https://doi.org/10.1155/2019/5262896>

Zhang, J., Lyu, T., Yang, Y., Wang, Y., Zheng, Y., Qu, S., Zhang, Z., Cai, X., Tang, C., & Huang, Y. (2021). Acupuncture at LR3 and KI3 shows a control effect on essential hypertension and targeted action on cerebral regions related to blood pressure regulation: A resting state functional magnetic resonance imaging study. *Acupuncture in Medicine*, *39*(1), 53-63.

Zhang, S., Wang, X., Yan, C. Q., Hu, S. Q., Huo, J. W., Wang, Z. Y., Zhou, P., Liu, C. H., & Liu, C. Z. (2018). Different mechanisms of contralateral- or ipsilateral-acupuncture to modulate the brain activity in patients with unilateral chronic shoulder pain: a pilot fMRI study. *J Pain Res*, *11*, 505-514. <https://doi.org/10.2147/JPR.S152550>

Zhang, Y., Wang, Z., Du, J., Liu, J., Xu, T., Wang, X., Sun, M., Wen, Y., Li, D., Liao, H., Zhao, Y., & Zhao, L. (2021). Regulatory Effects of Acupuncture on Emotional Disorders in Patients With Menstrual Migraine Without Aura: A Resting-State fMRI Study. *Front Neurosci*, *15*, 726505. <https://doi.org/10.3389/fnins.2021.726505>

Zhao, L., Liu, J., Zhang, F., Dong, X., Peng, Y., Qin, W., Wu, F., Li, Y., Yuan, K., von Deneen, K. M., Gong, Q., Tang, Z., & Liang, F. (2014). Effects of long-term acupuncture treatment on resting-state brain activity in migraine patients: a randomized controlled trial on active acupoints and inactive acupoints. *PLoS One*, *9*(6), e99538. <https://doi.org/10.1371/journal.pone.0099538>

Zhou, Y., Dai, A., Feng, S., Zhu, T., Liu, M., Shi, J., & Wang, D. (2024). Immediate neural effects of acupuncture manipulation time for stroke with motor dysfunction: a fMRI pilot study. *Frontiers in Neuroscience*, *17*, 1297149.
